# Different receptor models show differences in ligand binding strength and location: a computational drug screening for the tick-borne encephalitis virus

**DOI:** 10.1007/s11030-024-10850-8

**Published:** 2024-05-13

**Authors:** Felicitas Finke, Jonathan Hungerland, Ilia A. Solov’yov, Fabian Schuhmann

**Affiliations:** 1 Institute of Physics, Carl von Ossietzky Universität, Carl-von-Ossietzky-Str. 9-11, 26129 Oldenburg, Germany; 2 Research Centre for Neurosensory Science, Carl von Ossietzky Universität, Carl-von-Ossietzky-Str. 9-11, 26129 Oldenburg, Germany; 3 Center for Nanoscale Dynamics (CENAD), Carl von Ossietzky Universität, Ammerländer Heerstr. 114-118, 26129 Oldenburg, Germany; 4 Niels Bohr International Academy, Niels Bohr Institute, University of Copenhagen, Blegdamsvej 17, 2100 Copenhagen, Denmark

**Keywords:** Molecular dynamics, Drug discovery, Receptor models, AlphaFold

## Abstract

The tick-borne encephalitis virus (TBE) is a neurotrophic disease that has spread more rapidly throughout Europe and Asia in the past few years. At the same time, no cure or specific therapy is known to battle the illness apart from vaccination. To find a pharmacologically relevant drug, a computer-aided drug screening was initiated. Such a procedure probes a possible binding of a drug to the RNA Polymerase of TBE. The crystal structure of the receptor, however, includes missing and partially modeled regions, which rendered the structure incomplete and of questionable use for a thorough drug screening procedure. The quality of the receptor model was addressed by studying three putative structures created. We show that the choice of receptor models greatly influences the binding affinity of potential drug molecules and that the binding location could also be significantly impacted. We demonstrate that some drug candidates are unsuitable for one model but show decent results for another. Without any prejudice on the three employed receptor models, the study reveals the imperative need to investigate the receptor structure before drug binding is probed whether experimentally or computationally.

**Supplementary Information:**

The online version contains supplementary material available at 10.1007/s11030-024-10850-8.

## Introduction

The tick-borne encephalitis (TBE) virus is a neurotropical disease with flu-like symptoms that may evolve to a severe neurological illness. The virus can be subdivided into three main types, namely the European TBE (TBE-Eu), the Siberian TBE (TBE-Sib), and the far eastern variant (TBE-FE) [1], that differ by their death toll. Primarily, TBE-FE is associated with a severe course of the illness with long-lasting neurological effects [2] and a death toll of 20–60% of the infected. The other variants of the virus, TBE-Eu and TBE-Sib, exhibit a death rate of 1–2% and 6–8%, respectively [3]. However, the virus subtype alone is not the only criterion for a mild or severe case of the disease, as for each subtype any scenario is possible, and the implications are mainly influenced by the age or genetic predisposition of the patient [1].

The different TBE virus subclasses can be found in Europe and Asia and are classified as an epidemic in numerous countries. [2, 4, 5]. The disease associated with the TBE virus is notifiable in the European Union since 2012 [1]. According to the Annual Epidemiological Report of the ECDC (European Centre for Disease Prevention and Control), 24 countries within the European Economic Area reported a total of 3,734 cases of TBE in 2020 and an increase in the more recent years [2]. For instance, the reported cases increased by 30% in Germany in 2023 compared to 2022 [6].

The disease is mainly transmitted through tick bites [7]. In 1% of the cases, it is transmitted through consumption of products stemming from infected animals, like unpasteurized milk from cows, sheep, or goats. A case of a direct infection transmitted from human to human is not known [8]; however, a case has been reported in which the virus was transmitted through the transplantation of infected organs [9].

Even though the TBE virus is widely spread in Europe and Asia and studies have been performed to inhibit the similar Japanese encephalitis virus or the Zika virus [10–13], no medication is known to treat it, which reduces the options for supporting or symptomatic treatments, while effective vaccinations are available [1].

With rising global temperatures, the odds of ticks surviving winter increases, thereby boosting the chances of infection. The rising numbers highlight the need for specific medications that can be employed post-infection. Fortunately, nowadays suggestions for potentially pharmaceutically relevant drug candidates can be established computationally to screen a large number of potential drugs and find well-binding candidates prior to the individual experimental tests [14, 15]. The present investigation focuses on such a virtual drug screening procedure for the RNA-dependent polymerase (RdRp) protein of the TBE virus with the aim to find putative medicinally relevant drug ligands. A collection of 2,000 ligand molecules were investigated, similar to the frequently studied drug molecule that has the potency to inhibit the RdRp protein, namely the 7-Deaza-2’C-methyladenosine (7DMA); the 7DMA molecule has been documented to effect some viruses from the flavivirus genus [7].

The TBE virus contains an RdRp protein, which is essential for the replication of the virus inside the host system. The RdRp protein is highly conserved through various viruses of the flavivirus genus, which also includes the TBE virus. Such conservation might indicate the specific importance and sensitivity of the protein and thus reduces the chance that the virus can successfully undergo a sustainable mutation to gain immunity against the putative medications. Furthermore, it was shown in mice that the protein inhibits the growth of the neurites, which directly favors the onset of the neurological phase of the disease [16]. Lastly, the protein does not have a eucaryotic homologous counterpart. Therefore, a successful drug might have little to no secondary effects on human cells [7].

The RdRp protein structure is only partially available in its crystalline form with 98 out of 638 (partially) missing residues. The present study attempts to reconstruct the missing parts of the RdRp receptor through computational modeling. Three structural models were established and their differences and similarities rationalized. The first model employs the homology modeling approach relying on the SWISS-MODEL platform [17–21]. The second model employs AlphaFold [14, 22, 23] to reconstruct the structure of the entire receptor. The third model uses AlphaFold to replace only the partially modeled or missing residues, while keeping the rest of the crystal structure unchanged. The multiple models were considered to address the low-confidence regions in the receptor structure, such that the structure obtained using SWISS-MODEL could be compared to the two other models from the same virus genus. As computer-generated protein structures have warrying confidence levels in different regions, it is natural that no structure can be proven to be the only correct one.

The 2,000 potential drug ligands were then tested for all three models individually using the Automated Ligand Searcher (ALISE) [26], which is incorporated into the browser-based computational platform VIKING (Scandinavian online kit for nanoscale modeling) [27].

The most-promising ligands for all three models were compared in their binding energy and binding location. We have found that the atomistic details in the receptor structure severely influence the potential drug binding affinity and that it is unpractical to unquestioningly rely on a receptor structure obtained using any modeling tool. Three ligands were identified as the best-performing ligands in two of the three receptor models, making them the most robust independent of the receptor. While the results should be considered with care, an experimental study of these ligands might significantly further the cause in finding a medically relevant drug against the TBE virus.

## Background and methods

### The receptor structure

A potentially viable receptor for drug targeting and the inhibition of the TBE virus is its RNA-dependent RNA-polymerase (RdRp), which is essential for the self-replication of the virus. The RdRp protein is at the C-terminal of the so-called NS5 non-structural protein inside the virus, which consists of the methyltransferase domain followed by the polymerase domain [7, 16].

**Fig. 1:**
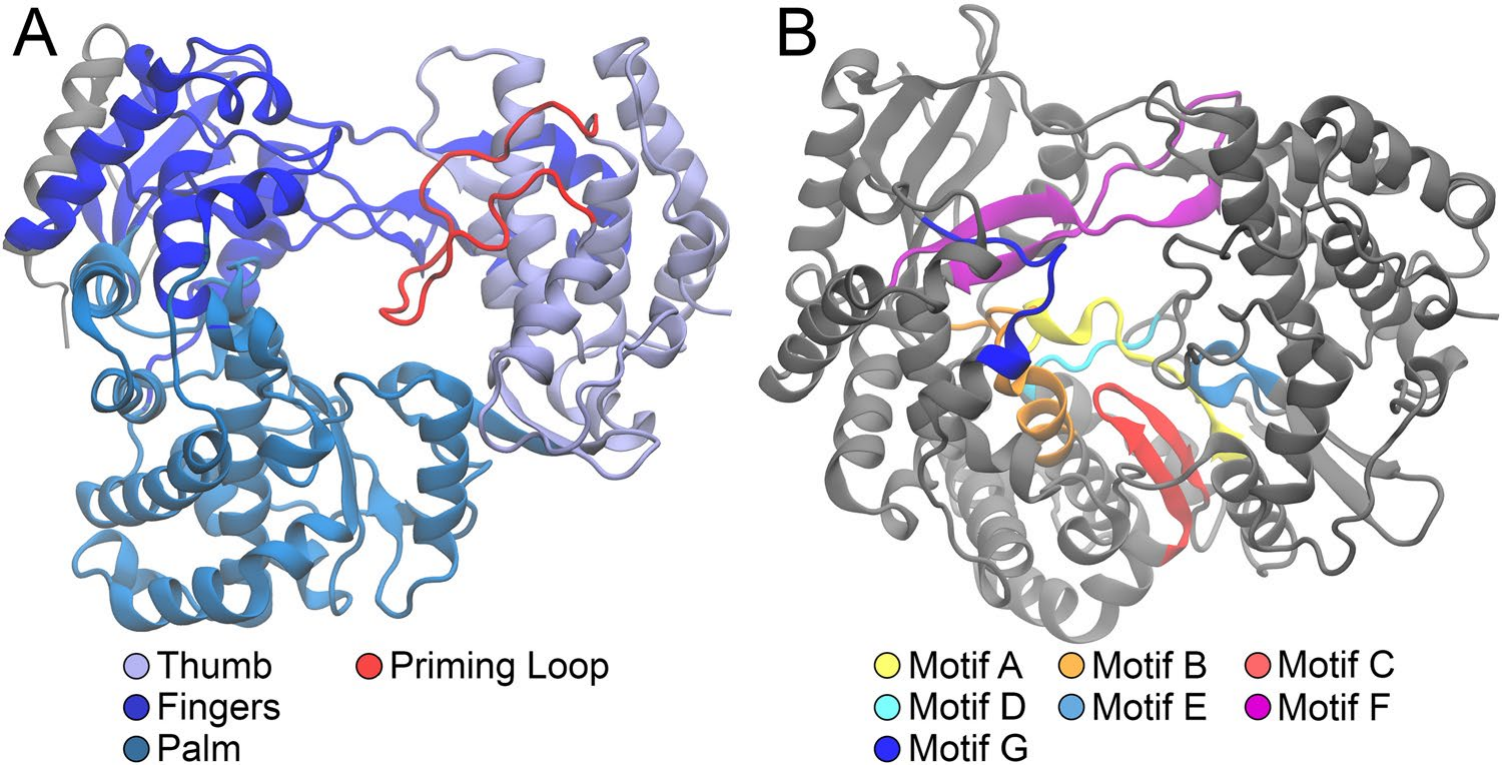
**A** Right-hand-architecture of the RdRp Protein of the TBE virus. The structure is subdivided into three regions surrounding the active site in its center. The so-called thumb domain is shown in gray-blue, the finger domain in blue, and the palm domain in teal colored. The priming loop (red) is part of the thumb domain. **B** Colored representation of the different motifs in the RdRp protein structure. Motifs A, B, C, and F interact directly with the so-called NTP. The other motifs are endowed with structural responsibilities

Figure 1 shows the major motifs in the structure of the RdRp protein of the TBE virus and its so-called right-hand architecture, which forms a catalytic unit. The structure is subdivided into three regions, surrounding the active site of the RdRp receptor. The thumb domain is shown in gray-blue, the finger domain in blue, and the palm domain in teal. The priming loop (red) is part of the thumb domain. Overall, the right-hand architecture can be further classified through the abundance of functional motifs, denoted as A–G in Fig. 1B. The motifs A, B, C, and F interact directly with the nucleoside triphosphate (NTP), which is the precursor for RNA synthesis.

The potential drug binding site of the RdRp protein is located in its active center, where the RNA transcription takes place. The priming loop limits the accessibility to the active site, so only ssRNA may enter the center during the initialization of the transcription [28, 29]. A well-binding drug ligand might, therefore, prohibit initialization of the RNA synthesis.

A crystal structure for the RdRp protein with the PDB ID 7D6N [28] is available in the PDB database [30]. The protein sequence comprises 638 amino acid residues, of which, in total, 19 residues at the N- and C-terminal have not been reconstructed. Furthermore, the crystal structure does not contain the well-defined positions of 50 amino acid residues and exhibits unmodeled side chains for 48 amino acid residues (see Table A1 in the Supplementary Material (SM)). As the partially modeled or missing residues are expected to be close to the active center, modeling methods were employed to obtain a more complete receptor structure.

Three different modeling methods were employed to generate the receptor structure. The first method uses the SWISS-MODEL [31] homology modeling, and the second utilizes the artificial intelligence workflow provided by AlphaFold [14, 22, 23]. Lastly, a hybrid model was considered, where the structural information obtained from AlphaFold was merged with the crystal structure, effectively replacing the missing and partially modeled regions.

The receptor obtained from SWISS-MODEL [17–21] requires the RdRp protein’s sequence and is denoted as Swiss. A visual inspection of the resulting receptor model showed some differences in crystal structures of similar viruses: the Japanese encephalitis virus (PDB ID 4K6M) [10] or the Zika virus (PDB ID 5TFR) [11], which belong to the same virus genus as the TBE virus. The Japanese encephalitis virus structure and the Zika virus exhibit less unmodeled residues and have been subjected to drug docking procedures in the past [12, 13].

**Fig. 2:**
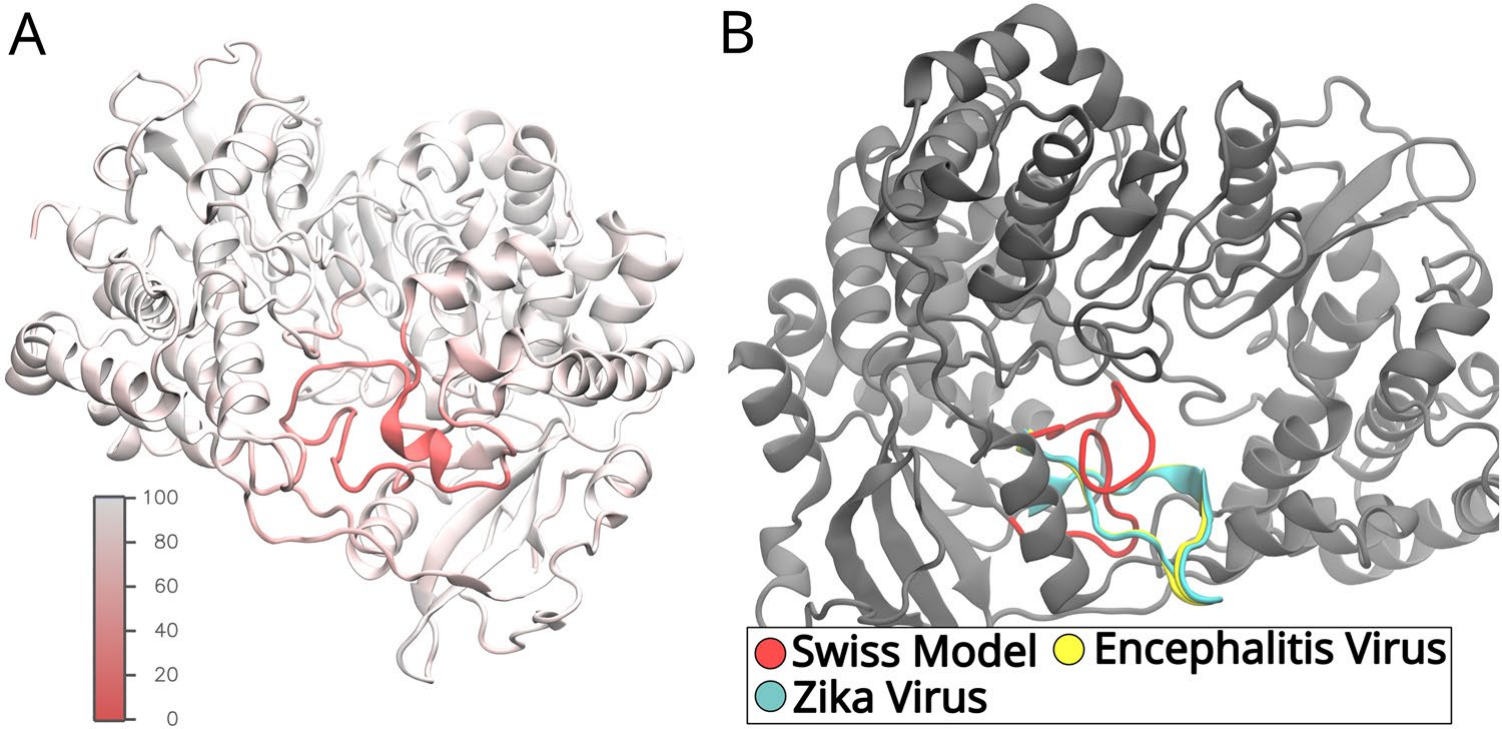
**A** The structure obtained from the Swiss model is colored according to the certainty estimated by the modeling process (arb. units). The certainty is measured on a scale from 0 to 100, with 0 describing an uncertain region and 100 denoting a high confidence. **B** The uncertain region obtained from the Swiss model is highlighted in red, while the same region is overlaid with the structure of the Japanese encephalitis virus (yellow) and the Zika virus (cyan)

A visual representation of the differences is shown in Fig. 2. With this in mind, different receptor models were employed to compare the drug ligand binding results. No judgement is made, however, on the realism of any of the resulting receptor models.

The second receptor was generated using AlphaFold [14, 22, 23]. AlphaFold only requires the amino acid sequence to generate a three-dimensional protein structure, but the generated structure can be refined by selected homologous crystal structures. In this case, the algorithm used four crystal structures for refinement (pdb IDs: 4C11 [24], 5U0C [25], 4K6M [10], 5U0B [25]). AlphaFold is an artificial intelligence tool trained on the existing crystal structures, which are, for instance, located in the PDB database. AlphaFold also generates the N- and C- terminal residues, which were completely unmodeled in the crystal structure. To match all studied models, these 19 amino acid residues were subsequently removed. Alpha denotes the receptor model obtained from AlphaFold.

Finally, because AlphaFold relies on the predictions from all available crystal structures, and not necessarily on the ones matching the RdRp protein of the TBE virus, a hybrid model was constructed, denoted by Hybrid. The model was obtained by aligning the crystal structure and Alpha model. The partially modeled or missing amino acid residues in the crystal structure were then inserted into the crystal structure from the Alpha model. The hybrid model, therefore, directly contains information from the crystal structure, supplemented by the information obtained with AlphaFold.

In the last step, the protonation states of the different amino acid residues had to be taken into account as, for instance, a wrongly protonated histidine residue can have significant effects on the binding affinity of molecules [32]. Considering a physiological pH-value of 7.4 [33], the so-called $\text{p}K_a$ values can be calculated, which was done employing the PROKKA3 program [34, 35] for the Swiss model. The resulting $\text{p}K_a$ values, see Table B1 in the Supplementary Materials, combined with a visual inspection, led to changes in the protonation of histidines. By default, all histidines were considered to be $\delta$-protonated, with only the His180 being $\varepsilon$-protonated. Furthermore, the Asp402 residue was additionally protonated. To keep the comparability of receptor models consistent, the protonation states from the Swiss model were also used for the Alpha and Hybrid models.

After the structures were modeled and the protonation states were set, a molecular dynamics (MD) simulation was conducted in multiple steps to equilibrate the structures. For the MD simulation, the VIKING platform was employed [27], which relies on the simulation software NAMD [36, 37] and VMD [38] for file preparation. All equilibration stages employed the CHARMM force field with CMAP corrections [39–41] with a temperature of 310 K. The systems were neutralized, and sodium chloride ions were added to achieve a salt concentration of 0.15 mol/L. In the first two stages, the MD simulation was conducted in an NPT (constant number of atoms, pressure, and temperature) ensemble with a timestep of 1 fs and a pressure of 1.01325 bar. Everything but the solvent was restrained in the first stage, while in the second stage, the amino acid residue’s side chains were allowed to move. The first stage was initiated with 1000 conjugate gradient minimization steps. Stages three and four were conducted in the NVT (constant number of atoms, volume, and temperature) ensemble. In the third stage, no restraints on atomic positions were imposed. Finally, in stage 4, the integrator time step was set to 2 fs, and the covalent bond to hydrogen atoms were considered rigid. The receptor protein structures were simulated for 30 ns in stage 4. For the Hybrid equilibration, a second minimization step was introduced to account for the artificially over-long bonds generated through the manual combination of Alpha and the crystal structures.

The final snapshots from the equilibration simulation were further used for the drug screening workflow.

### Automated ligand searcher

All three receptor models of the RdRp protein were used in the ALISE program [26] to predict possible drug-like ligands that could bind inside the receptor. ALISE is an automated workflow incorporated into the VIKING online platform [27], which sorts ligands based on their binding affinity to a provided receptor model. A search space to limit the docking to physiologically sensible binding sites can also be defined which was chosen to be the active center in the middle of the receptor structures. Different ligand molecules were generated by ALISE that was provided with the SMILES string of 7DMA, a known drug for viruses similar to the TBE virus. The search space for the drug docking was chosen to contain the RNA and the NTP binding location to block the most important functions of the receptor. With this information, ALISE automatically found 2000 similar ligands according to the Tanimoto score in the PubChem database [26]. The Tanimoto score *K* is given for two molecular fingerprints by [42]

1 $$\begin{aligned} K=\frac{|A\cap B|}{|A\cup B|}. \end{aligned}$$

ALISE relies on a three-step process in which, gradually, potential ligands are filtered out, while the computational accuracy of the binding prediction increases. Drug docking is performed in the first step relying on the docking program Vina (AutoDock Vina 1.1.2)[43] , with ligands being prepared by the AutoDockTools [44] and Openbabel [45] programs. The docked ligands were then sorted according to their binding scores. The highest-ranked 30 ligands of the docking phase were then considered in the second step of ALISE relying on atomistic MD simulations.

The MD simulation phase includes simulations of the receptor, the ligand, and a complex consisting of the receptor and ligand in an implicit solvent [26, 46]. While the docking procedure in the first step is static, the MD simulations allow motions in the receptor and ligand. These motions, for instance, might lead to a repulsion and force ligands to leave the binding pocket, which is a clear indication of a low binding affinity. The binding energy of the ligands was estimated from the three different simulations per ligand (receptor only, ligand only, and complex simulations), and the ligands were sorted by their binding affinity. ALISE employs the molecular mechanics-generalized Born and surface area continuum solvation (MM/GBSA) method [46]. ALISE’s MD step employs NAMD [36, 37], while the simulation files were prepared using VMD [38]. The Charmm General Force Field (cgenFF) [47] was employed for the ligands, while the receptor was modeled using the Charmm Force Field with CMAP corrections [40, 48–54].

The five highest-scoring ligands from the MD step were subjected to a free energy perturbation (FEP) stage. In the FEP stage, windowed MD simulations were conducted in which the interactions between the receptor and the ligand were gradually decoupled and recoupled. Conformational restraints are used and later subtracted to avoid a diffusion of the ligand when the interactions are completely decoupled. Such an approach allows the most precise estimate for the binding free energy and establishes the final ranking of the remaining ligands [26]. A total of 50 simulation windows were employed, each including an MD equilibration. The FEP step employs NAMD [36, 37] with an explicit solvent and the same force fields and parameters as used in the MD step described above.

## Results

### Stability

**Fig. 3:**
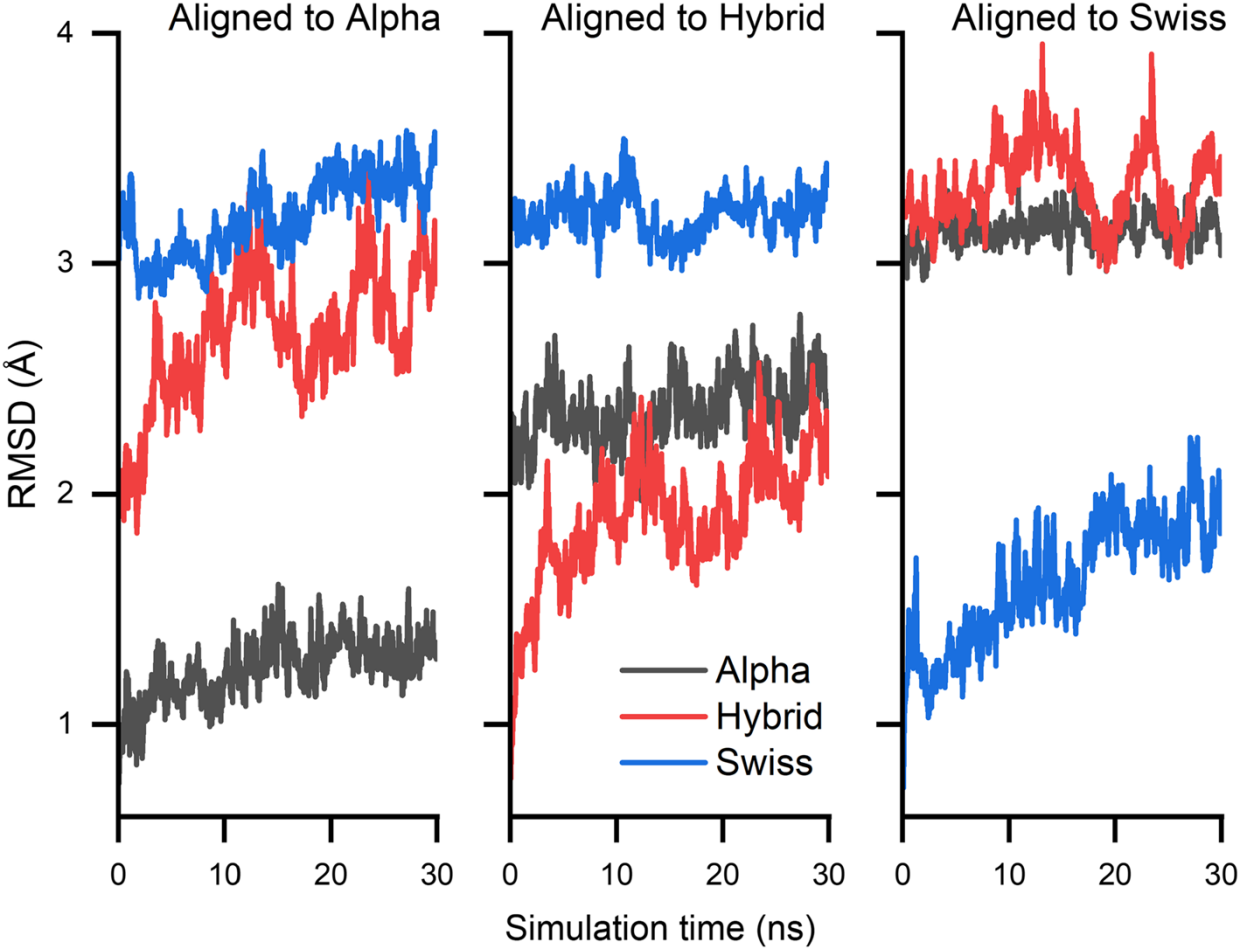
The three panels show the time evolution of the RMSD for the whole protein during the 30 ns equilibration phase. For each panel, structures were aligned to the respective reference structures prior to RMSD calculation, i.e., in the first panel, all three simulation trajectories are aligned to the first snapshot of the Alpha model simulation. The dependencies show an overall stable behavior of the different receptor models with most fluctuations in the Hybrid model. Throughout the simulation, one also notices that the Swiss model differs more from the Alpha model than the Hybrid model and that the Hybrid model and the Alpha model are similarly dissimilar to the Swiss model, concurring with the expectations

Following the initial equilibration MD simulations, the stabilities of the structures were validated by employing the Root Mean Square Deviation (RMSD) analysis. The RMSD values for the last snapshot of the equilibration simulations are compiled in Table 1 to compare the different structures with each other. The RMSD was additionally calculated for the regions with high and low certainties, following the structure prediction based on the Swiss model. A visual representation of the confidence is shown in Fig. 2A. The resulting values suggest that the three receptor structures are relatively similar with each other in the high-certainty regions, while Swiss and Alpha models differ significantly in the uncertainty regions. Additionally, the RMSD time evolution for the entire protein is shown in Fig. 3. The overall low RMSD below 4 Å for the protein consisting of 619 residues indicates fairly stable structures [55–59]. Additionally, even though the magnitude of the fluctuations is higher in the Hybrid and Swiss model compared to the Alpha model, they seem to evolve around a flat plateau after 10 ns or 20 ns, respectively.

**Table 1:** The root mean square deviation (RMSD) between the final configurations of the different receptor models

Reference simulation	Swiss compared to hybrid (Å)	Swiss compared to Alpha (Å)	Hybrid compared to Alpha (Å)
Whole protein	3.93	4.24	3.66
Low certainty	12.19	12.78	4.81
High certainty	3.04	3.35	3.60

The RMSD is calculated for the whole protein, high-certainty, and low-certainty regions as estimated by the SWISS model platform [17] during receptor generation. In this table, the high-certainty regions have a confidence score above 50; low certainty are the regions below 50 as seen in Fig. 2A. All receptor models are similar in the high-certainty regions, while significant differences are found primarily between Swiss and Alpha models in the low-certainty regions

### Drug screening

Following the stability analysis of the different receptor models, the virtual screening program ALISE was used to study the binding affinity of a variety of ligands. The three steps of ALISE were conducted automatically, starting with the drug docking stage. All stages were completed for the three studied receptor models. After each step, ALISE ranks the ligands; the best-performing ligands are then advanced to the next stage:

The ranking for the best 50 docked ligands in their five best binding positions is provided in Tables C2–C4 in the Supplementary Material. The 30 best-performing ligands were considered for the MD stage. During the MD stage, all atoms in the system are free to move and exhibit attraction and repulsion interactions. These interactions might result in the ligand to leave the originally determined binding pocket. Therefore, additionally to a more rigorous estimate of the binding energy, ligands that propelled out of their binding pocket can be excluded from further testing. The different locations of the potential ligands obtained from the docking stage provide different starting configurations for the MD simulations.

The binding free energy estimated from the MD simulations for all receptors and all ligands is summarized in the Supplementary Material (see Tables D5–D7 in Supplementary Material). A visual inspection of the bound ligands in the different receptors reveals three noteworthy results.

**Fig. 4:**
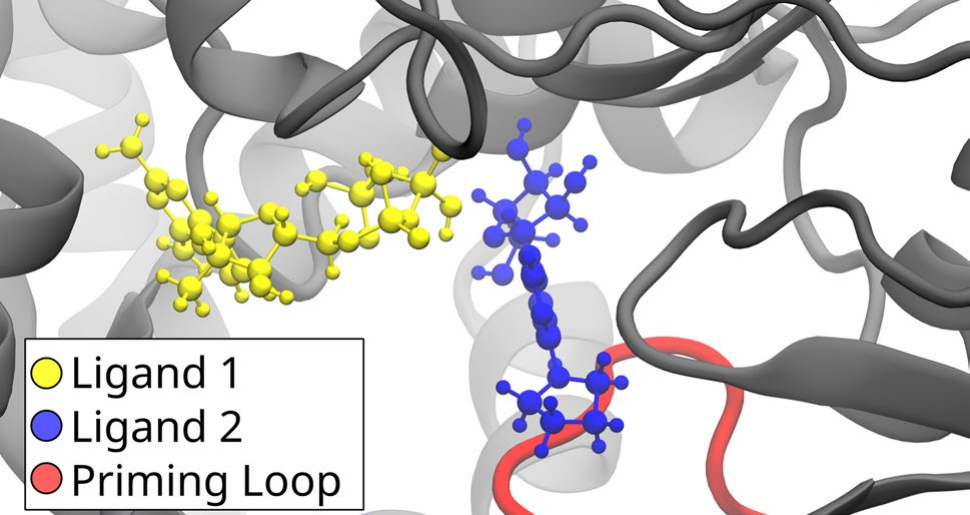
Two ligands are shown within the Hybrid model receptor, with the priming loop in red. Ligand 1 (yellow) is the only ligand that obtained a negative binding energy in the MD stage, while the other 10 best-performing ligands, represented by the exemplary ligand 2 (blue), perform more poorly

In the course of completed MD simulations, most ligands were able to stay in their originally placed binding locations as determined by the docking stage. The original binding locations may differ, however, even within a single receptor model. Figure 4 shows the positioning of the second-ranked ligand (yellow) compared to an exemplary ligand (blue), which represents the orientations of the majority of the 10 best-performing ligands bound in the Hybrid model. During the MD stage, it is revealed that only the yellow ligand results in a favorable binding energy. Thus, the ALISE’s MD step corrects unphysical placements of ligands during the docking stage.

**Fig. 5:**
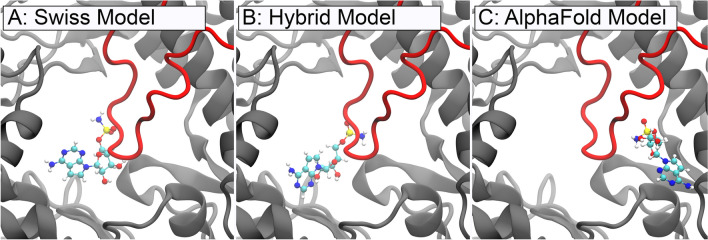
Binding locations for the ligand with the PubChem ID 189237 within the different receptor models. In the Alpha model, the same ligand exhibits a different binding site on the other side of the priming loop compared to the Swiss and Hybrid Models

Another example is provided by the ligand with the PubChem ID 189237 which was ranked among the best 30 performing ligands in the docking stage for all three receptors; however, its placement inside the receptors turned out to be different. The ligand was thus subjected to MD simulations three times, once for each receptor model. Figure 5 shows the ligand’s positioning in the different receptor models. The Swiss and Hybrid model are consistent with each other in respect to the ligand binding location, but not orientation. The Alpha model features the ligand on the opposite side of the priming loop.

**Fig. 6:**
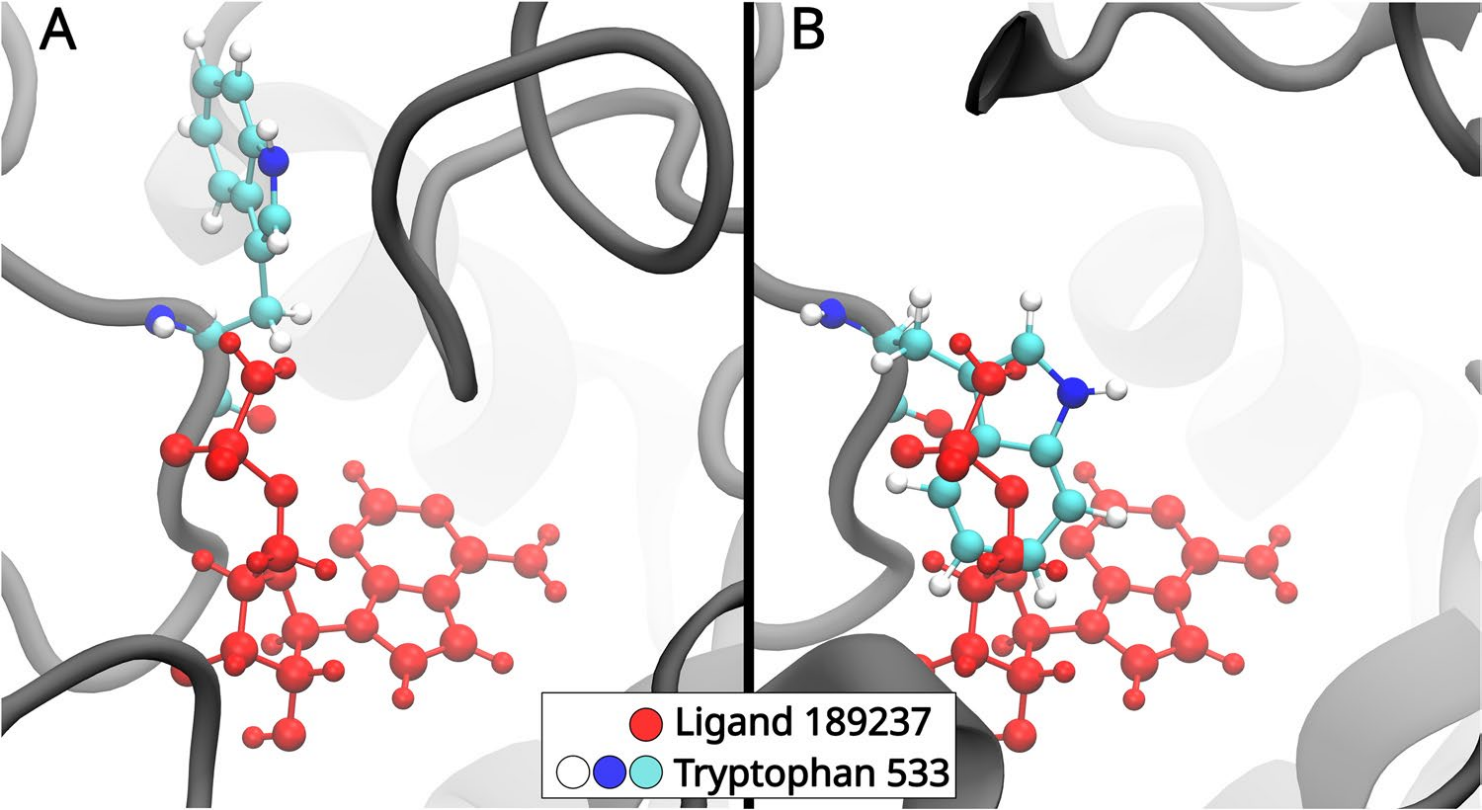
Orientation of the tryptophan 533 residue in the Swiss model (**A**) and the Alpha model (**B**). The panels also show the placement of the ligand with the PubChem ID 189237 in the Swiss model. The difference in orientation of the tryptophan side chain perturbs the potential binding site of the ligand, making it impossible to bind to that particular location. The slight change in side chain orientation might, therefore, significantly affect the ligand binding site

Another result followed from the visual inspection of conformations of the final snapshot of the different MD simulations. Here, the Trp533 residue in the Swiss and Alpha model receptor was studied, which turned out to assume two different distinct orientations as visualized in Fig. 6. It turns out that the side chain orientation, as seen in the Alpha model, actively perturbs the potential drug binding location, as has been identified, for example, for the ligand with the PubChem ID 189237, seen in Fig. 6B. Figure 6A shows the orientation of the tryptophan in the Swiss model with the bound ligand. The slight change in side chain orientation might, therefore, significantly affect the binding site.

**Fig. 7:**
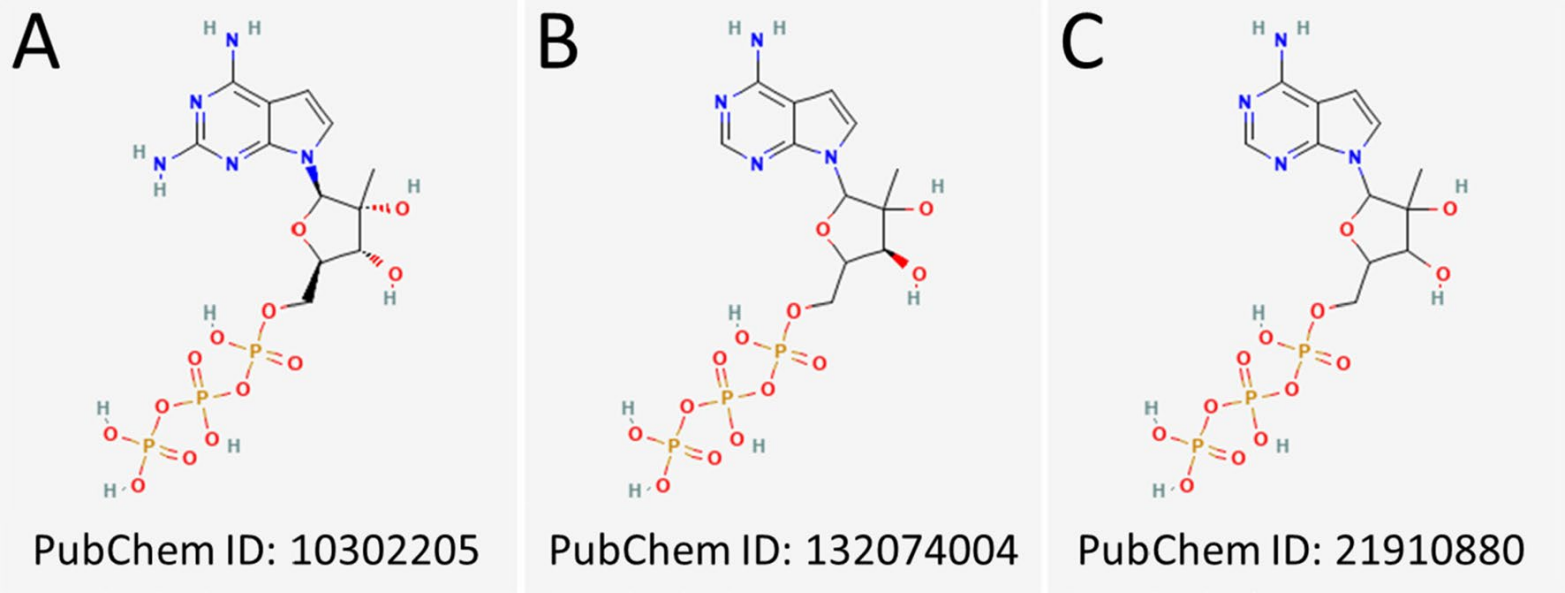
Structural formulas for the three most robust ligands that show favorable binding across the different receptor models. The corresponding PubChem IDs are indicated. The ligands in **B** and **C** are structurally identical; however, the ligand in (**B**) is an enantiomer of the ligand in (**C**)

Five ligands with the best binding energy estimates from the MD simulations were subjected to the FEP calculations. Remarkably, not a single ligand had simultaneously a favorable (negative) binding free energy for all the three receptor models. However, three ligands still showed binding affinity for two receptor structures. The chemical structure of these three ligands is illustrated in Fig. 7. The binding free energies estimated by the FEP stage are given in Table 2. The ligands occurring in two receptor models are colored according to their pair. Complete tables containing individual contributions can be found in the Supplementary Material (see Tables E8–E10 in Supplementary Material). To validate the binding locations of the three reoccurring ligands, additional MD simulations were performed for the highlighted receptor-ligand complexes in three replicas each. Based on an RMSD analysis, the ligands maintain their location in the receptor structure for at least another 100 ns. A time evolution of the RMSD for the validation simulations is found in Fig. F11.

### Comparison of high-ranking ligands

**Fig. 8:**
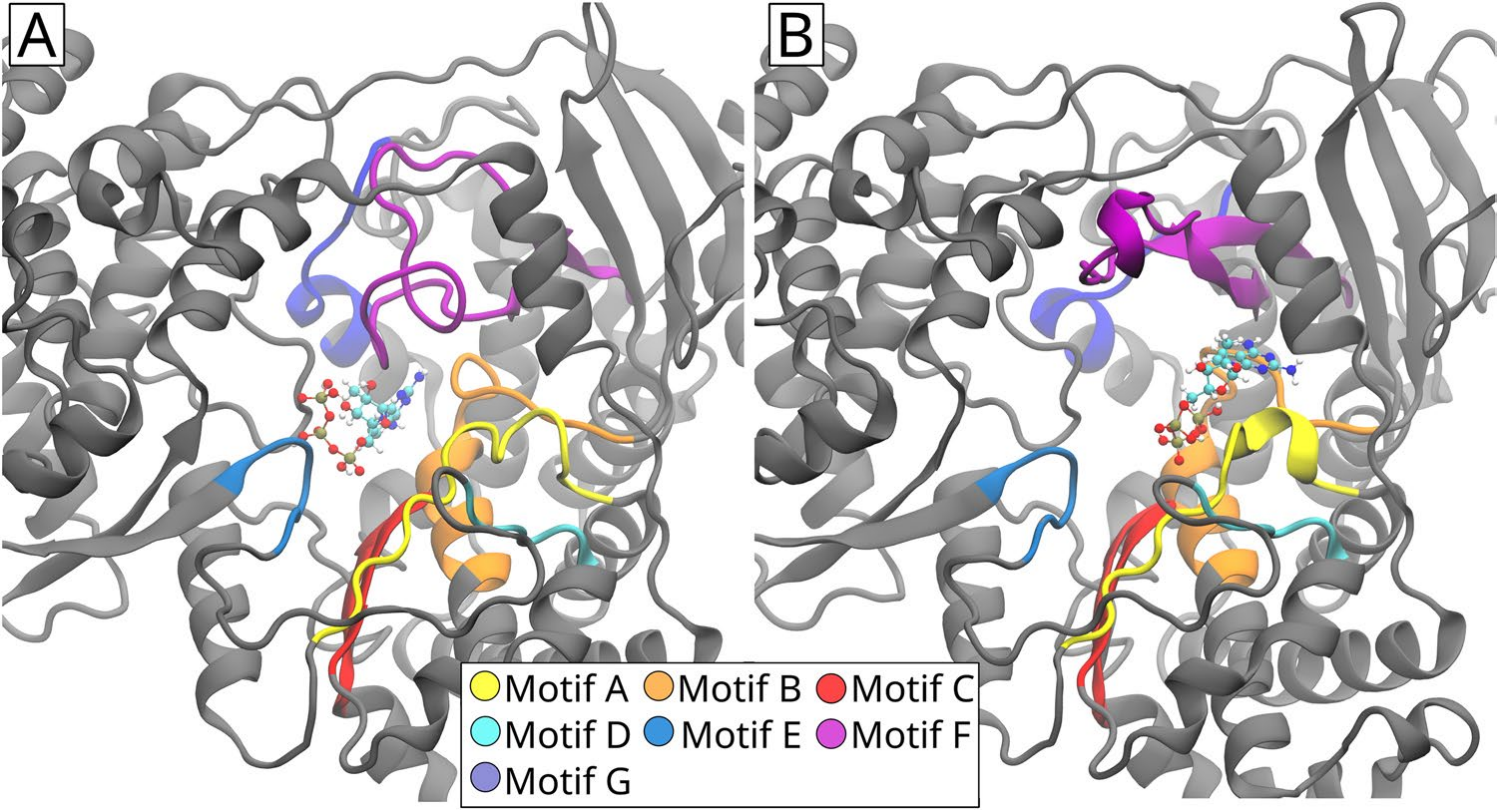
Positioning of the ligand with the PubChem ID 10302205 inside the Swiss model (**A**) and the Hybrid model (**B**). In the Swiss model, the ligand is located in the center of the active site, potentially interacting with the B, C, E, and F motifs. In the Hybrid model, the ligand is close to the A, B, C, and F motifs

Differences in the starting configuration for the FEP calculations carry over from the MD simulations. As such, the binding location may differ within the three ligands. For instance, the ligand with the PubChem ID 10302205 (rank four in the Swiss model, rank two in the Hybrid model) exhibits a slightly different orientation in the Swiss and Hybrid models, respectively; a visual inspection shows that the ligand might prohibit the ssRNA from entering the structure of the receptor in the Swiss model. At the same time, the ligand’s location might indicate an inhibition of the NTP interaction in the Hybrid model. As NTP is a precursor to synthesizing RNA, blocking its interaction site makes the ligand a likely inhibitor for the virus. More precisely, in the Swiss model, the ligand is exposed to a large surface area of the motifs B and F and parts of the motifs C and E of the receptor, see Fig. 1B. In the Hybrid model, the ligand is close to the A, B, C, and F motifs. Figure 8 shows a visualization of the drug binding location and orientation at the beginning of the FEP calculations. The A, B, C, and F motifs interact directly with the NTP. Therefore, one can suspect that the ligand may block the binding site for the NTP and significantly influence the functionality of the RdRp. Furthermore, the ligand binds fairly robustly with a binding free energy of negative 13–16 kcal/mol to both receptor models (see Table 2).

**Table 2:**
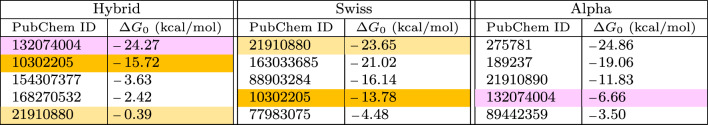
The binding free energies obtained from the FEP stage in the ALISE workflow are shown for the different receptor models

The ligands are indicated by their PubChem ID. The highlighted ligands ranked among the best-performing five ligands in the MD stage for two different receptor models. A detailed account of binding free energy estimates is given in the Supplementary Material (Tables E8–E10 in Supplementary Material)

The ligand with the Pubchem ID 132074004 is ranked first in the Hybrid model and fourth in the Alpha model. The ligand has a similar position in both receptor models, but a different orientation. While the triphosphate region is oriented in different directions, the ligand’s aromatic rings share a similar location. The ligand is close to the A, B, C, and F motifs, similar to the binding site exhibited in Fig. 8B. Hence, the ligand might also inhibit NTP interactions.

Lastly, the ligand with the PubChem ID 21910880, which ranks highest in the Swiss model, was the least performing ligand in the Hybrid model with a binding free energy estimate of − 0.39 kcal/mol. The binding energy value close to zero indicates a weak binding and small perturbations might already lead to the drug molecule’s detachment. Additionally, the ligand is located in the poor confidence region of the receptor with the greatest structural variability. The ligand is, however, an enantiomer on the base of the previous ligand with the Pubchem ID 132074004; in other words, they are configurationally isomeric.

Considering that the ligands 132074004 and 21910880 are structurally similar, the racemat (21910880) is within the best five performing ligands for all three receptor models, which makes it the most robust ligand that potentially binds to all three versions of the receptor structure.

## Conclusion

The choice of receptor structure is essential for the quality of a computational ligand screening experiment. We have demonstrated that differences in the secondary structure within receptor models of the RNA-depended RNA-polymerase of the tick-borne encephalitis virus and minor differences like the orientation of a single side chain may drastically affect the binding of drug ligands. The structural differences directly influence the size of binding pockets, the orientation and location of bound ligands, and, thus, the effect a ligand might have on the protein’s functionality.

We have computationally constructed three different receptor structures with different modeling tools and compared their susceptibility to 2000 potential drug molecules. From the docking stage, the 50 best-performing ligands advanced to the molecular dynamics stage. While 17 of these best-performing ligands could be found across all three different receptor models, no ligand was among the best-performing ligands for all the three investigated structures after the MD stage. The results indicate that without endowing any level of realism on neither the Swiss Model-generated structure, nor the AlphaFold-generated structure, nor their hybrid variant, special care should be taken as a single model could lead to inconsistent results and should not be taken as the all-truth without further study. Additionally, all ligands could be compared regarding their physiochemical properties or their closest residues in the different receptor structures after each stage in ALISE’s virtual screening workflow, which would go beyond a study focusing on important structural differences to drug binding in receptor structures.

As a silver lining, however, the ligand with the PubChem ID 21910880 turned out to be structurally identical to the ligand with the PubChem ID 132074004, except for a mirrored oxygen. If one considers these two highly similar compounds, a single potential drug molecule is identified which is robust over all three different receptor models and binds in all of them. Thus, it might be feasible to test the ligand with the PubChem ID 132074004 experimentally, as its performance is close to independent of the structure model. The ligand with the PubChem ID 10302205 performs robustly in two of the three models. In summary, without trusting the physiological realism of the receptor structures, the ligands 10302205 and 21910880 might be feasible candidates for further testing as potential treatments of the TBE.

Overall, trust in a receptor structure is hard earned. We demonstrated the effects of differences in receptors on the binding of drug molecules. Therefore, the results of virtual screening can only be trusted as far as the receptor structure model.

## Supplementary Information

Below is the link to the electronic supplementary material.

Supplementary file 1 (pdf 493 KB)

## Data Availability

Data supporting the findings of this study are available upon reasonable request. Requests for access to the data should be directed to Ilia A. Solov’yov (ilia.solovyov@uni-oldenburg.de). The data are not publicly available due to the amount of data and licensing on third party software for non-academic users.

## Code availability

All used programs are cited. Some programs contain proprietary elements, which are not free for non-academic users.
