# Supplementary material for: Different receptor models show differences in ligand binding strength and location: a computational drug screening for the tick-borne encephalitis virus
Source: Mol Divers. 2024 May 13;29(1):281–92. doi: 10.1007/s11030-024-10850-8 (PMC11785706; doi:10.1007/s11030-024-10850-8)
Supplement: Supplementary file 1 — (pdf 493 KB) [file 11030_2024_10850_MOESM1_ESM.pdf]

Appendix A Missing or partially modelled residues

Table A1: Sequence position of the missing or partially modeled amino acids. The third column indicates whether the residue was added in the Hybrid model.

Amino Acid Index	Status	Edited
264	missing	not added
265	missing	not added
266	missing	not added
267	missing	not added
268	missing	not added
269	missing	not added
270	missing	not added
271	partially modeled	added
272	partially modeled	added
276	partially modeled	added
280	partially modeled	added
281	partially modeled	added
297	partially modeled	added
298	partially modeled	added
311	partially modeled	added
327	partially modeled	added
336	partially modeled	added
337	partially modeled	added
338	partially modeled	added
341	partially modeled	added
344	partially modeled	added
354	partially modeled	added
357	partially modeled	added
358	partially modeled	added
359	partially modeled	added
388	partially modeled	added
398	partially modeled	added

Continuation on next page

Table A1 – Continuation from previous page

**Amino Acid Index**	**Status**	**Edited**
405	partially modeled	added
408	missing	added
409	missing	added
410	missing	added
411	missing	added
412	missing	added
413	missing	added
414	missing	added
415	missing	added
416	missing	added
419	partially modeled	added
421	missing	added
424	partially modeled	added
425	partially modeled	added
434	partially modeled	added
456	missing	added
457	missing	added
458	missing	added
459	missing	added
460	missing	added
461	missing	added
462	missing	added
463	missing	added
464	missing	added
465	missing	added
466	missing	added
467	missing	added
468	missing	added
469	missing	added
470	missing	added
471	missing	added
472	missing	added
473	missing	added
474	missing	added
475	missing	added
476	partially modeled	added
477	partially modeled	added
614	partially modeled	added
637	partially modeled	added
638	partially modeled	added
646	partially modeled	added
677	partially modeled	added
734	partially modeled	added
744	missing	added
751	partially modeled	added

Continuation on next page

Table A1 – Continuation from previous page

**Amino Acid Index**	**Status**	**Edited**
793	partially modeled	added
808	partially modeled	added
829	partially modeled	added
835	partially modeled	added
842	partially modeled	added
849	partially modeled	added
852	partially modeled	added
855	partially modeled	added
857	partially modeled	added
859	partially modeled	added
862	partially modeled	added
877	partially modeled	added
881	partially modeled	added
889	partially modeled	added
890	missing	not added
891	missing	not added
892	missing	not added
893	missing	not added
894	missing	not added
895	missing	not added
896	missing	not added
897	missing	not added
898	missing	not added
899	missing	not added
900	missing	not added
901	missing	not added

Appendix B pK_a values

Table B1: Calculated pK_a values for amino acids which can be (de-)protonated. The amino acid type, index and pK_a values are listed according to their location in the NS5 of the TBE virus.

**Amino Acid**	**Index**	**pK_a**	**Model-pK_a**
ASP	277	1.76	3.8
ASP	296	3.38	3.8
ASP	338	3.19	3.8
ASP	346	2.36	3.8
ASP	361	1.93	3.8
ASP	380	4.94	3.8
ASP	415	3.6	3.8
ASP	429	3.94	3.8
ASP	437	3.96	3.8
ASP	496	3.16	3.8
ASP	534	3.35	3.8
ASP	535	5.17	3.8
ASP	540	6.05	3.8
ASP	547	0.34	3.8
ASP	550	4.53	3.8
ASP	587	3.85	3.8
ASP	593	3.85	3.8
ASP	599	3.01	3.8
ASP	635	4.09	3.8
ASP	650	3.75	3.8
ASP	664	3.5	3.8
ASP	665	7.15	3.8
ASP	672	2.48	3.8
ASP	673	3.07	3.8
ASP	684	4.05	3.8
ASP	691	2.89	3.8
ASP	721	1.88	3.8
ASP	731	4.13	3.8
ASP	733	4.68	3.8
ASP	772	2.32	3.8
ASP	787	3.8	3.8
ASP	809	2.54	3.8
ASP	812	3.97	3.8
ASP	821	3.88	3.8
ASP	836	2.95	3.8
ASP	845	3.79	3.8
ASP	882	3.28	3.8
ASP	888	3.58	3.8
GLU	275	4.57	4.5

Continuation on next page

Table B1 – Continuation from previous page

**Amino Acid**	**Index**	**pK_a**	**Model-pK_a**
GLU	280	4.02	4.5
GLU	287	4.47	4.5
GLU	291	4.59	4.5
GLU	298	4.52	4.5
GLU	337	4.57	4.5
GLU	358	4.5	4.5
GLU	366	3.87	4.5
GLU	384	4.54	4.5
GLU	398	4.68	4.5
GLU	399	2.26	4.5
GLU	416	4.96	4.5
GLU	425	4.29	4.5
GLU	428	4.42	4.5
GLU	438	4.8	4.5
GLU	439	4.6	4.5
GLU	441	4.55	4.5
GLU	460	4.26	4.5
GLU	465	4.63	4.5
GLU	486	6.85	4.5
GLU	488	4.81	4.5
GLU	495	4.31	4.5
GLU	509	5.93	4.5
GLU	527	4.46	4.5
GLU	549	4.87	4.5
GLU	551	5.54	4.5
GLU	552	5.25	4.5
GLU	559	4.57	4.5
GLU	561	4.24	4.5
GLU	626	4.92	4.5
GLU	628	3.43	4.5
GLU	632	4.59	4.5
GLU	645	4.64	4.5
GLU	653	3.38	4.5
GLU	654	4.35	4.5
GLU	694	4.67	4.5
GLU	696	4.32	4.5
GLU	705	4.37	4.5
GLU	706	4.78	4.5
GLU	716	3.81	4.5
GLU	734	5.4	4.5
GLU	751	3.19	4.5
GLU	808	2.9	4.5
GLU	829	4.55	4.5
GLU	833	4.61	4.5
GLU	856	4.73	4.5

Continuation on next page

Table B1 – Continuation from previous page

**Amino Acid**	**Index**	**pK_a**	**Model-pK_a**
GLU	859	3.67	4.5
GLU	869	3.81	4.5
GLU	878	4.19	4.5
HIS	294	6.43	6.5
HIS	299	6.63	6.5
HIS	443	5.52	6.5
HIS	450	5.73	6.5
HIS	497	6.73	6.5
HIS	562	5.6	6.5
HIS	575	3.93	6.5
HIS	637	6.88	6.5
HIS	651	6.25	6.5
HIS	697	6.08	6.5
HIS	712	3.92	6.5
HIS	713	5.92	6.5
HIS	715	6.04	6.5
HIS	769	2.65	6.5
HIS	799	4.97	6.5
HIS	826	6.3	6.5
HIS	844	6.24	6.5
CYS	395	11.44	9
CYS	448	10.89	9
CYS	451	11.43	9
CYS	590	10.21	9
CYS	666	13.08	9
CYS	710	12.96	9
CYS	729	10.84	9
CYS	745	9.52	9
CYS	754	10.23	9
CYS	781	11.7	9
CYS	848	9.77	9
CYS	886	10.08	9
TYR	289	10.58	10
TYR	301	15.04	10
TYR	306	10.52	10
TYR	310	12.15	10
TYR	453	14.51	10
TYR	477	11.67	10
TYR	515	12.06	10
TYR	519	10.23	10
TYR	532	10.72	10
TYR	557	10.66	10
TYR	574	16.76	10
TYR	609	13.17	10
TYR	680	10.45	10

Continuation on next page

Table B1 – Continuation from previous page

**Amino Acid**	**Index**	**pK_a**	**Model-pK_a**
TYR	759	12.14	10
TYR	767	13	10
TYR	839	11.83	10
TYR	883	14.11	10
LYS	272	11.38	10.5
LYS	274	10.43	10.5
LYS	276	12.42	10.5
LYS	327	10.27	10.5
LYS	357	11.11	10.5
LYS	359	10.52	10.5
LYS	363	10.36	10.5
LYS	372	11.42	10.5
LYS	389	10.11	10.5
LYS	391	10.42	10.5
LYS	403	11.37	10.5
LYS	405	10.59	10.5
LYS	424	10.2	10.5
LYS	458	10.08	10.5
LYS	461	9.43	10.5
LYS	462	8.9	10.5
LYS	470	12.67	10.5
LYS	521	11.37	10.5
LYS	542	9.02	10.5
LYS	563	10.16	10.5
LYS	572	10.07	10.5
LYS	577	6.64	10.5
LYS	580	9.07	10.5
LYS	618	12.44	10.5
LYS	687	8.86	10.5
LYS	690	11.56	10.5
LYS	720	11.37	10.5
LYS	757	9.08	10.5
LYS	828	10.41	10.5
LYS	830	10.53	10.5
LYS	842	10.36	10.5
LYS	855	10.44	10.5
LYS	862	11.4	10.5
LYS	870	10.23	10.5
LYS	873	10.45	10.5
LYS	879	10.58	10.5
LYS	881	10.92	10.5
ARG	281	12.19	12.5
ARG	286	12.04	12.5
ARG	297	12.45	12.5
ARG	302	12.41	12.5

Continuation on next page

Table B1 – Continuation from previous page

**Amino Acid**	**Index**	**pK_a**	**Model-pK_a**
ARG	311	12.41	12.5
ARG	336	13.2	12.5
ARG	341	13.39	12.5
ARG	354	12.44	12.5
ARG	376	13.41	12.5
ARG	385	12.78	12.5
ARG	388	12.57	12.5
ARG	393	15.35	12.5
ARG	397	11.96	12.5
ARG	419	12.3	12.5
ARG	440	13.47	12.5
ARG	442	12.5	12.5
ARG	447	12.26	12.5
ARG	459	10.74	12.5
ARG	473	11.36	12.5
ARG	483	10.24	12.5
ARG	501	10.91	12.5
ARG	556	12.53	12.5
ARG	583	14.31	12.5
ARG	586	12.43	12.5
ARG	597	12.91	12.5
ARG	598	12.37	12.5
ARG	601	13.22	12.5
ARG	623	11.69	12.5
ARG	640	12.39	12.5
ARG	643	12.58	12.5
ARG	646	12.22	12.5
ARG	649	12.49	12.5
ARG	655	13.14	12.5
ARG	658	12.21	12.5
ARG	669	11.79	12.5
ARG	674	13.45	12.5
ARG	677	12.93	12.5
ARG	689	11.08	12.5
ARG	723	13.51	12.5
ARG	730	12.11	12.5
ARG	738	11.42	12.5
ARG	740	10.98	12.5
ARG	750	12.42	12.5
ARG	770	12.99	12.5
ARG	771	12.87	12.5
ARG	774	10.25	12.5
ARG	793	11.62	12.5
ARG	816	13.8	12.5
ARG	835	12.73	12.5

Continuation on next page

Table B1 – Continuation from previous page

**Amino Acid**	**Index**	**p**K_a	**Model-p**K_a
ARG	854	12.1	12.5
ARG	857	12.59	12.5
ARG	872	13.09	12.5
ARG	889	12.31	12.5
C-	889	3.18	3.2

Appendix C   Docking Stage Results

Table C2: Results for the docking stage in ALISE for the AlphaFold receptor model. The table contains the best five binding configuration for each ligand, which are identified by their PubChem ID.

Rank	Binding Position	Ligand	Binding energy kcal/mol
1	1	132073976	-8.0
	2	132073976	-7.8
	3	132073976	-7.7
	4	132073976	-7.6
	5	132073976	-7.6
2	1	21910890	-7.7
	2	21910890	-7.2
	3	21910890	-7.1
	4	21910890	-7.0
	5	21910890	-6.9
3	1	89442359	-7.5
	2	89442359	-6.9
	3	89442359	-6.8
	4	89442359	-6.8
	5	89442359	-6.8
4	1	132074005	-7.3
	2	132074005	-6.7
	3	132074005	-6.3
	4	132074005	-6.3
	5	132074005	-6.2
5	1	71171599	-7.2
	2	71171599	-7.1
	3	71171599	-7.0
	4	71171599	-7.0
	5	71171599	-6.9
6	1	78173475	-7.2
	2	78173475	-6.7
	3	78173475	-6.7
	4	78173475	-6.5
	5	78173475	-6.4
7	1	10164932	-7.1
	2	10164932	-6.9
	3	10164932	-6.8
	4	10164932	-6.8
	5	10164932	-6.7
8	1	131965796	-7.1
	2	131965796	-6.5
	3	131965796	-6.4

Continuation on next page

Table C2 – Continuation from previous page

Rank	Binding Position	Ligand	Binding energy kcal/mol
	4	131965796	-6.3
	5	131965796	-5.9
9	1	142377395	-7.1
	2	142377395	-6.4
	3	142377395	-6.3
	4	142377395	-6.2
	5	142377395	-6.0
10	1	70911224	-7.1
	2	70911224	-6.7
	3	70911224	-6.5
	4	70911224	-6.4
	5	70911224	-6.4
11	1	126970220	-7.0
	2	126970220	-6.4
	3	126970220	-5.9
	4	126970220	-5.8
	5	126970220	-5.7
12	1	131965793	-7.0
	2	131965793	-6.6
	3	131965793	-6.5
	4	131965793	-6.5
	5	131965793	-6.5
13	1	131965829	-7.0
	2	131965829	-6.4
	3	131965829	-6.3
	4	131965829	-5.8
	5	131965829	-5.8
14	1	131965842	-7.0
	2	131965842	-6.5
	3	131965842	-6.2
	4	131965842	-6.0
	5	131965842	-5.9
15	1	132074004	-7.0
	2	132074004	-6.8
	3	132074004	-6.7
	4	132074004	-6.3
	5	132074004	-6.2
16	1	168287218	-7.0
	2	168287218	-6.7
	3	168287218	-6.4
	4	168287218	-6.3
	5	168287218	-6.3
17	1	189237	-7.0
	2	189237	-6.8

Continuation on next page

Table C2 – Continuation from previous page

Rank	Binding Position	Ligand	Binding energy kcal/mol
	3	189237	-6.8
	4	189237	-6.8
	5	189237	-6.7
18	1	275781	-7.0
	2	275781	-6.2
	3	275781	-6.2
	4	275781	-6.1
	5	275781	-6.1
19	1	3012941	-7.0
	2	3012941	-6.9
	3	3012941	-6.9
	4	3012941	-6.7
	5	3012941	-6.6
20	1	71229422	-7.0
	2	71229422	-6.4
	3	71229422	-6.0
	4	71229422	-5.8
	5	71229422	-5.7
21	1	88903284	-7.0
	2	88903284	-6.9
	3	88903284	-6.8
	4	88903284	-6.8
	5	88903284	-6.7
22	1	92163236	-7.0
	2	92163236	-6.2
	3	92163236	-6.1
	4	92163236	-6.0
	5	92163236	-6.0
23	1	92237025	-7.0
	2	92237025	-6.3
	3	92237025	-6.2
	4	92237025	-6.1
	5	92237025	-6.1
24	1	10107793	-6.9
	2	10107793	-6.1
	3	10107793	-6.0
	4	10107793	-5.7
	5	10107793	-5.7
25	1	10302154	-6.9
	2	10302154	-6.8
	3	10302154	-6.7
	4	10302154	-6.7
	5	10302154	-6.6
26	1	126970231	-6.9

Continuation on next page

Table C2 – Continuation from previous page

Rank	Binding Position	Ligand	Binding energy kcal/mol
	2	126970231	-6.3
	3	126970231	-6.2
	4	126970231	-6.0
	5	126970231	-6.0
27	1	131965777	-6.9
	2	131965777	-6.0
	3	131965777	-6.0
	4	131965777	-5.9
	5	131965777	-5.7
28	1	131965837	-6.9
	2	131965837	-6.3
	3	131965837	-6.1
	4	131965837	-6.1
	5	131965837	-6.0
29	1	131965850	-6.9
	2	131965850	-6.2
	3	131965850	-6.2
	4	131965850	-6.1
	5	131965850	-5.6
30	1	21910968	-6.9
	2	21910968	-6.9
	3	21910968	-6.8
	4	21910968	-6.8
	5	21910968	-6.7
31	1	77983075	-6.9
	2	77983075	-6.7
	3	77983075	-6.7
	4	77983075	-6.5
	5	77983075	-6.4
32	1	89099561	-6.9
	2	89099561	-6.6
	3	89099561	-6.4
	4	89099561	-6.2
	5	89099561	-6.2
33	1	90692422	-6.9
	2	90692422	-6.8
	3	90692422	-6.6
	4	90692422	-6.5
	5	90692422	-6.2
34	1	91201335	-6.9
	2	91201335	-6.2
	3	91201335	-6.1
	4	91201335	-6.1
	5	91201335	-6.1

Continuation on next page

Table C2 – Continuation from previous page

Rank	Binding Position	Ligand	Binding energy kcal/mol
35	1	102439955	-6.8
	2	102439955	-6.6
	3	102439955	-6.5
	4	102439955	-6.3
	5	102439955	-6.2
36	1	123150745	-6.8
	2	123150745	-6.4
	3	123150745	-5.6
	4	123150745	-5.6
	5	123150745	-5.6
37	1	126970232	-6.8
	2	126970232	-6.1
	3	126970232	-5.7
	4	126970232	-5.6
	5	126970232	-5.6
38	1	131965799	-6.8
	2	131965799	-6.8
	3	131965799	-6.5
	4	131965799	-6.5
	5	131965799	-6.3
39	1	141664882	-6.8
	2	141664882	-6.7
	3	141664882	-6.6
	4	141664882	-6.6
	5	141664882	-6.5
40	1	142617626	-6.8
	2	142617626	-6.0
	3	142617626	-5.9
	4	142617626	-5.9
	5	142617626	-5.9
41	1	156126051	-6.8
	2	156126051	-5.9
	3	156126051	-5.8
	4	156126051	-5.8
	5	156126051	-5.6
42	1	156627111	-6.8
	2	156627111	-6.4
	3	156627111	-6.0
	4	156627111	-6.0
	5	156627111	-6.0
43	1	168270532	-6.8
	2	168270532	-6.4
	3	168270532	-6.3
	4	168270532	-6.3

Continuation on next page

Table C2 – Continuation from previous page

Rank	Binding Position	Ligand	Binding energy kcal/mol
	5	168270532	-6.1
44	1	308234	-6.8
	2	308234	-6.3
	3	308234	-6.3
	4	308234	-6.2
	5	308234	-6.2
45	1	90812394	-6.8
	2	90812394	-6.1
	3	90812394	-6.0
	4	90812394	-6.0
	5	90812394	-6.0
46	1	91033965	-6.8
	2	91033965	-6.6
	3	91033965	-6.6
	4	91033965	-6.5
	5	91033965	-6.4
47	1	92237027	-6.8
	2	92237027	-6.7
	3	92237027	-6.5
	4	92237027	-6.3
	5	92237027	-6.3
48	1	9943250	-6.8
	2	9943250	-6.4
	3	9943250	-6.2
	4	9943250	-6.1
	5	9943250	-6.0
49	1	10302205	-6.7
	2	10302205	-6.6
	3	10302205	-6.6
	4	10302205	-6.6
	5	10302205	-6.5
50	1	123219605	-6.7
	2	123219605	-6.6
	3	123219605	-6.4
	4	123219605	-6.2
	5	123219605	-6.0

Table C3: Results for the docking stage in ALISE for the Hybrid receptor model. The table contains the best five binding configuration for each ligand, which are identified by their PubChem ID.

Rank	Binding Position	Ligand	Binding energy kcal/mol
1	1	88903284	-9.1
	2	88903284	-8.5
	3	88903284	-8.4
	4	88903284	-8.4
	5	88903284	-8.3
2	1	10302154	-8.0
	2	10302154	-7.8
	3	10302154	-7.6
	4	10302154	-7.5
	5	10302154	-7.4
3	1	23518335	-7.8
	2	23518335	-7.2
	3	23518335	-7.2
	4	23518335	-7.1
	5	23518335	-6.9
4	1	71171599	-7.8
	2	71171599	-7.4
	3	71171599	-7.1
	4	71171599	-7.0
	5	71171599	-7.0
5	1	21910890	-7.7
	2	21910890	-7.4
	3	21910890	-7.0
	4	21910890	-6.9
	5	21910890	-6.8
6	1	10164932	-7.6
	2	10164932	-7.4
	3	10164932	-7.3
	4	10164932	-7.2
	5	10164932	-7.1
7	1	10302205	-7.6
	2	10302205	-7.4
	3	10302205	-7.4
	4	10302205	-7.3
	5	10302205	-7.2
8	1	3012941	-7.6
	2	3012941	-7.4
	3	3012941	-7.1
	4	3012941	-7.0
	5	3012941	-6.9
9	1	132074005	-7.5

Continuation on next page

Table C3 – Continuation from previous page

Rank	Binding Position	Ligand	Binding energy kcal/mol
	2	132074005	-7.4
	3	132074005	-7.3
	4	132074005	-7.2
	5	132074005	-7.1
10	1	77983075	-7.4
	2	77983075	-7.2
	3	77983075	-7.0
	4	77983075	-6.8
	5	77983075	-6.6
11	1	89442359	-7.3
	2	89442359	-7.3
	3	89442359	-7.1
	4	89442359	-7.0
	5	89442359	-7.0
12	1	132074004	-7.2
	2	132074004	-7.2
	3	132074004	-7.1
	4	132074004	-7.1
	5	132074004	-7.0
13	1	141664882	-7.2
	2	141664882	-7.1
	3	141664882	-7.1
	4	141664882	-7.1
	5	141664882	-7.0
14	1	21910880	-7.2
	2	21910880	-7.2
	3	21910880	-7.1
	4	21910880	-7.0
	5	21910880	-7.0
15	1	70911224	-7.2
	2	70911224	-7.0
	3	70911224	-6.7
	4	70911224	-6.7
	5	70911224	-6.6
16	1	72562976	-7.2
	2	72562976	-6.9
	3	72562976	-6.8
	4	72562976	-6.4
	5	72562976	-6.3
17	1	92249433	-7.2
	2	92249433	-7.1
	3	92249433	-7.0
	4	92249433	-7.0
	5	92249433	-6.6

Continuation on next page

Table C3 – Continuation from previous page

Rank	Binding Position	Ligand	Binding energy kcal/mol
18	1	168270532	-7.1
	2	168270532	-6.7
	3	168270532	-6.7
	4	168270532	-6.7
	5	168270532	-6.6
19	1	168287218	-7.1
	2	168287218	-6.7
	3	168287218	-6.7
	4	168287218	-6.5
	5	168287218	-6.4
20	1	22217204	-7.1
	2	22217204	-7.0
	3	22217204	-6.7
	4	22217204	-6.7
	5	22217204	-6.4
21	1	10107793	-7.0
	2	10107793	-6.8
	3	10107793	-6.7
	4	10107793	-6.3
	5	10107793	-6.3
22	1	117669199	-7.0
	2	117669199	-6.8
	3	117669199	-6.5
	4	117669199	-6.5
	5	117669199	-6.4
23	1	189237	-7.0
	2	189237	-6.9
	3	189237	-6.9
	4	189237	-6.9
	5	189237	-6.6
24	1	46185996	-7.0
	2	46185996	-6.7
	3	46185996	-6.7
	4	46185996	-6.7
	5	46185996	-6.6
25	1	90692422	-7.0
	2	90692422	-7.0
	3	90692422	-6.8
	4	90692422	-6.6
	5	90692422	-6.4
26	1	91201335	-7.0
	2	91201335	-6.8
	3	91201335	-6.7
	4	91201335	-6.4

Continuation on next page

Table C3 – Continuation from previous page

Rank	Binding Position	Ligand	Binding energy kcal/mol
	5	91201335	-6.3
27	1	131881832	-6.9
	2	131881832	-6.7
	3	131881832	-6.6
	4	131881832	-6.6
	5	131881832	-6.6
28	1	154307377	-6.9
	2	154307377	-6.7
	3	154307377	-6.6
	4	154307377	-6.6
	5	154307377	-6.6
29	1	158533254	-6.9
	2	158533254	-6.0
	3	158533254	-6.0
	4	158533254	-5.8
	5	158533254	-5.8
30	1	23518336	-6.9
	2	23518336	-6.7
	3	23518336	-6.7
	4	23518336	-6.6
	5	23518336	-6.4
31	1	59998678	-6.9
	2	59998678	-6.5
	3	59998678	-6.4
	4	59998678	-6.1
	5	59998678	-6.1
32	1	71199518	-6.9
	2	71199518	-6.8
	3	71199518	-6.7
	4	71199518	-6.6
	5	71199518	-6.6
33	1	78173475	-6.9
	2	78173475	-6.9
	3	78173475	-6.9
	4	78173475	-6.8
	5	78173475	-6.7
34	1	89099561	-6.9
	2	89099561	-6.7
	3	89099561	-6.5
	4	89099561	-6.5
	5	89099561	-6.4
35	1	89127354	-6.9
	2	89127354	-6.8
	3	89127354	-6.7

Continuation on next page

Table C3 – Continuation from previous page

**Rank**	**Binding Position**	**Ligand**	**Binding energy kcal/mol**
	4	89127354	-6.4
	5	89127354	-6.3
36	1	92249434	-6.9
	2	92249434	-6.7
	3	92249434	-6.7
	4	92249434	-6.7
	5	92249434	-6.4
37	1	9835419	-6.9
	2	9835419	-6.8
	3	9835419	-6.4
	4	9835419	-6.4
	5	9835419	-6.4
38	1	102439955	-6.8
	2	102439955	-6.3
	3	102439955	-6.3
	4	102439955	-6.3
	5	102439955	-6.2
39	1	10753174	-6.8
	2	10753174	-6.3
	3	10753174	-6.2
	4	10753174	-6.0
	5	10753174	-6.0
40	1	117669200	-6.8
	2	117669200	-6.6
	3	117669200	-6.6
	4	117669200	-6.5
	5	117669200	-6.4
41	1	132073976	-6.8
	2	132073976	-6.6
	3	132073976	-6.5
	4	132073976	-6.5
	5	132073976	-6.5
42	1	132560563	-6.8
	2	132560563	-6.8
	3	132560563	-6.7
	4	132560563	-6.6
	5	132560563	-6.4
43	1	163750378	-6.8
	2	163750378	-6.3
	3	163750378	-6.2
	4	163750378	-6.2
	5	163750378	-6.1
44	1	21774277	-6.8
	2	21774277	-6.4

Continuation on next page

Table C3 – Continuation from previous page

Rank	Binding Position	Ligand	Binding energy kcal/mol
	3	21774277	-6.3
	4	21774277	-6.2
	5	21774277	-6.0
45	1	21854257	-6.8
	2	21854257	-6.3
	3	21854257	-6.2
	4	21854257	-6.1
	5	21854257	-6.1
46	1	21910877	-6.8
	2	21910877	-6.5
	3	21910877	-6.1
	4	21910877	-6.1
	5	21910877	-6.0
47	1	21910968	-6.8
	2	21910968	-6.6
	3	21910968	-6.6
	4	21910968	-6.5
	5	21910968	-6.5
48	1	275781	-6.8
	2	275781	-6.7
	3	275781	-6.7
	4	275781	-6.6
	5	275781	-6.5
49	1	3007920	-6.8
	2	3007920	-6.7
	3	3007920	-6.3
	4	3007920	-6.3
	5	3007920	-6.3
50	1	46936593	-6.8
	2	46936593	-6.7
	3	46936593	-6.4
	4	46936593	-6.3
	5	46936593	-6.1

Table C4: Results for the docking stage in ALISE for the Swiss receptor model. The table contains the best five binding configuration for each ligand, which are identified by their PubChem ID.

Rank	Binding Position	Ligand	Binding energy kcal/mol
1	1	132074004	-8.7
	2	132074004	-8.5
	3	132074004	-8.2
	4	132074004	-8.1
	5	132074004	-7.9
2	1	141664882	-8.6
	2	141664882	-8.2
	3	141664882	-8.2
	4	141664882	-8.1
	5	141664882	-8.1
3	1	23518335	-8.3
	2	23518335	-8.2
	3	23518335	-8.0
	4	23518335	-7.7
	5	23518335	-7.6
4	1	71171599	-8.3
	2	71171599	-7.9
	3	71171599	-7.9
	4	71171599	-7.8
	5	71171599	-7.6
5	1	10164932	-8.2
	2	10164932	-8.1
	3	10164932	-7.9
	4	10164932	-7.9
	5	10164932	-7.9
6	1	3012941	-8.2
	2	3012941	-8.2
	3	3012941	-8.2
	4	3012941	-7.9
	5	3012941	-7.8
7	1	21910880	-8.1
	2	21910880	-8.1
	3	21910880	-8.0
	4	21910880	-7.9
	5	21910880	-7.8
8	1	88903284	-8.1
	2	88903284	-8.0
	3	88903284	-7.8
	4	88903284	-7.8
	5	88903284	-7.8
9	1	89442359	-8.1

Continuation on next page

Table C4 – Continuation from previous page

**Rank**	**Binding Position**	**Ligand**	**Binding energy kcal/mol**
	2	89442359	-7.9
	3	89442359	-7.8
	4	89442359	-7.8
	5	89442359	-7.7
10	1	92244160	-8.1
	2	92244160	-7.4
	3	92244160	-7.1
	4	92244160	-7.1
	5	92244160	-6.9
11	1	53633108	-8.0
	2	53633108	-7.2
	3	53633108	-7.1
	4	53633108	-7.0
	5	53633108	-6.9
12	1	132073976	-7.9
	2	132073976	-7.6
	3	132073976	-7.6
	4	132073976	-7.6
	5	132073976	-7.4
13	1	163033685	-7.9
	2	163033685	-7.7
	3	163033685	-7.6
	4	163033685	-7.5
	5	163033685	-7.4
14	1	21910890	-7.9
	2	21910890	-7.5
	3	21910890	-7.4
	4	21910890	-7.2
	5	21910890	-7.0
15	1	10302205	-7.8
	2	10302205	-7.7
	3	10302205	-7.6
	4	10302205	-7.6
	5	10302205	-7.5
16	1	10302154	-7.7
	2	10302154	-7.7
	3	10302154	-7.6
	4	10302154	-7.5
	5	10302154	-7.4
17	1	126970231	-7.7
	2	126970231	-7.0
	3	126970231	-6.8
	4	126970231	-6.6
	5	126970231	-6.6

Continuation on next page

Table C4 – Continuation from previous page

Rank	Binding Position	Ligand	Binding energy kcal/mol
18	1	156627125	-7.7
	2	156627125	-7.4
	3	156627125	-7.1
	4	156627125	-6.8
	5	156627125	-6.7
19	1	189237	-7.7
	2	189237	-7.4
	3	189237	-7.3
	4	189237	-7.3
	5	189237	-7.3
20	1	70911224	-7.7
	2	70911224	-7.3
	3	70911224	-7.2
	4	70911224	-7.2
	5	70911224	-7.2
21	1	77983075	-7.7
	2	77983075	-7.4
	3	77983075	-7.3
	4	77983075	-7.1
	5	77983075	-6.9
22	1	92237025	-7.7
	2	92237025	-7.6
	3	92237025	-7.3
	4	92237025	-7.1
	5	92237025	-7.0
23	1	92237026	-7.7
	2	92237026	-7.6
	3	92237026	-7.4
	4	92237026	-7.3
	5	92237026	-7.2
24	1	92237032	-7.7
	2	92237032	-7.1
	3	92237032	-7.0
	4	92237032	-7.0
	5	92237032	-6.9
25	1	102439955	-7.6
	2	102439955	-7.1
	3	102439955	-7.1
	4	102439955	-6.9
	5	102439955	-6.9
26	1	156126049	-7.6
	2	156126049	-7.3
	3	156126049	-7.2
	4	156126049	-6.6

Continuation on next page

Table C4 – Continuation from previous page

Rank	Binding Position	Ligand	Binding energy kcal/mol
	5	156126049	-6.6
27	1	156627111	-7.6
	2	156627111	-7.5
	3	156627111	-7.2
	4	156627111	-6.8
	5	156627111	-6.8
28	1	168311672	-7.6
	2	168311672	-7.3
	3	168311672	-7.2
	4	168311672	-6.8
	5	168311672	-6.8
29	1	44628581	-7.6
	2	44628581	-6.6
	3	44628581	-6.6
	4	44628581	-6.4
	5	44628581	-6.3
30	1	71229422	-7.6
	2	71229422	-6.7
	3	71229422	-6.7
	4	71229422	-6.6
	5	71229422	-6.5
31	1	89442364	-7.6
	2	89442364	-6.8
	3	89442364	-6.8
	4	89442364	-6.8
	5	89442364	-6.7
32	1	90692422	-7.6
	2	90692422	-7.4
	3	90692422	-6.9
	4	90692422	-6.8
	5	90692422	-6.7
33	1	92249433	-7.6
	2	92249433	-6.9
	3	92249433	-6.9
	4	92249433	-6.9
	5	92249433	-6.9
34	1	154307377	-7.5
	2	154307377	-7.5
	3	154307377	-7.2
	4	154307377	-7.1
	5	154307377	-7.1
35	1	156513844	-7.5
	2	156513844	-7.1
	3	156513844	-7.0

Continuation on next page

Table C4 – Continuation from previous page

Rank	Binding Position	Ligand	Binding energy kcal/mol
	4	156513844	-6.9
	5	156513844	-6.8
36	1	168270532	-7.5
	2	168270532	-7.1
	3	168270532	-7.1
	4	168270532	-7.1
	5	168270532	-7.0
37	1	275781	-7.5
	2	275781	-6.8
	3	275781	-6.8
	4	275781	-6.7
	5	275781	-6.6
38	1	308234	-7.5
	2	308234	-7.1
	3	308234	-6.9
	4	308234	-6.9
	5	308234	-6.8
39	1	46185996	-7.5
	2	46185996	-7.5
	3	46185996	-7.2
	4	46185996	-7.1
	5	46185996	-7.0
40	1	60012722	-7.5
	2	60012722	-6.8
	3	60012722	-6.7
	4	60012722	-6.6
	5	60012722	-6.6
41	1	78173475	-7.5
	2	78173475	-7.2
	3	78173475	-7.1
	4	78173475	-7.0
	5	78173475	-7.0
42	1	130330316	-7.4
	2	130330316	-7.3
	3	130330316	-6.8
	4	130330316	-6.7
	5	130330316	-6.7
43	1	140862995	-7.4
	2	140862995	-7.2
	3	140862995	-6.8
	4	140862995	-6.7
	5	140862995	-6.7
44	1	156126051	-7.4
	2	156126051	-7.2

Continuation on next page

Table C4 – Continuation from previous page

Rank	Binding Position	Ligand	Binding energy kcal/mol
	3	156126051	-6.8
	4	156126051	-6.6
	5	156126051	-6.4
45	1	22217204	-7.4
	2	22217204	-7.3
	3	22217204	-7.2
	4	22217204	-7.1
	5	22217204	-7.0
46	1	275452	-7.4
	2	275452	-7.3
	3	275452	-7.1
	4	275452	-7.1
	5	275452	-7.0
47	1	46936593	-7.4
	2	46936593	-7.0
	3	46936593	-6.8
	4	46936593	-6.6
	5	46936593	-6.6
48	1	57401651	-7.4
	2	57401651	-7.3
	3	57401651	-7.0
	4	57401651	-6.9
	5	57401651	-6.9
49	1	70826341	-7.4
	2	70826341	-7.3
	3	70826341	-7.2
	4	70826341	-7.2
	5	70826341	-7.2
50	1	71199518	-7.4
	2	71199518	-7.2
	3	71199518	-6.9
	4	71199518	-6.9
	5	71199518	-6.7

Appendix D Molecular Dynamics Stage Results

Table D5: Results for the MD stage in ALISE for the AlphaFold receptor model. The ligands are identified by their PubChem ID. The total binding energy estimate is provided as well as the individual contributions from the Coulomb force, van der Waals (vdW) forces and covalent bonds.

Ligand	Coulomb	vdW	Binding	Total
189237	-90.39	-28.64	26.15	-92.88
275781	-15.07	-81.60	11.04	-85.63
21910890	-62.37	-36.65	16.74	-82.27
132074004	-30.20	-70.86	21.59	-79.47
89442359	-66.92	-41.18	28.71	-79.39
126970231	-48.18	-43.19	12.85	-78.52
3012941	-64.34	-60.74	49.00	-76.09
71229422	-46.44	-38.63	12.20	-72.87
10302154	-18.16	-73.34	19.92	-71.58
168287218	-29.96	-68.49	27.63	-70.82
131965777	-41.39	-60.06	30.89	-70.56
70911224	-50.50	-49.04	30.10	-69.44
131965837	-0.75	-90.55	22.60	-68.70
92163236	-33.46	-62.43	27.34	-68.55
126970220	-52.15	-43.45	27.11	-68.49
142377395	-3.75	-83.83	19.64	-67.94
10164932	-27.19	-78.71	38.07	-67.82
131965793	-39.58	-61.62	39.97	-61.24
131965850	-11.06	-55.92	9.93	-57.05
71171599	-17.84	-74.40	35.65	-56.58
132073976	-22.81	-46.88	15.56	-54.14
131965796	-2.58	-69.83	19.58	-52.82
131965842	-26.99	-62.22	37.70	-51.50
10107793	-29.11	-48.28	27.01	-50.38
78173475	-22.57	-50.27	22.96	-49.89
92237025	-22.70	-53.75	28.10	-48.35
131965829	-8.45	-55.92	18.56	-45.81

Table D6: Results for the MD stage in ALISE for the Hybrid receptor model. The ligands are identified by their PubChem ID. The total binding energy estimate is provided as well as the individual contributions from the Coulomb force, van der Waals (vdW) forces and covalent bonds.

**Ligand**	**Coulomb**	**vdW**	**Binding**	**Total**
168270532	25.02	-48.23	-10.52	-33.73
10302205	52.10	-75.57	-8.10	-31.58
154307377	17.50	-39.70	1.90	-20.31
21910880	16.09	-31.13	-3.81	-18.85
132074004	26.06	-50.43	6.85	-17.52
189237	-2.07	-21.78	6.42	-17.42
92249433	33.16	-53.37	4.22	-15.99
91201335	13.55	-43.86	20.13	-10.19
46185996	48.37	-53.35	-4.25	-9.22
71171599	19.90	-29.14	1.95	-7.29
10302154	22.70	-46.60	21.58	-2.32
168287218	23.10	-40.71	15.80	-1.81
77983075	30.16	-38.62	7.09	-1.37
158533254	5.83	-8.84	3.37	0.36
3012941	51.70	-51.03	5.74	6.40
23518335	24.95	-32.30	17.21	9.85
21910890	30.21	-30.79	12.18	11.60
10107793	-32.46	29.91	14.66	12.11
72562976	30.58	-19.59	3.95	14.95
89442359	17.77	-4.17	1.82	15.43
22217204	40.95	-39.52	14.43	15.85
70911224	23.20	-30.40	27.38	20.17
131881832	7.77	-5.66	21.83	23.95
10164932	30.31	-0.99	-1.03	28.30

Table D7: Results for the MD stage in ALISE for the Swiss receptor model. The ligands are identified by their PubChem ID. The total binding energy estimate is provided as well as the individual contributions from the Coulomb force, van der Waals (vdW) forces and covalent bonds.

**Ligand**	**Coulomb**	**vdW**	**Binding**	**Total**
163033685	-48.73	-49.20	-0.06	-98.00
21910880	-3.61	-81.00	-8.33	-92.94
77983075	-39.64	-29.90	-12.46	-82.00
10302205	-15.20	-71.48	5.52	-81.17
88903284	-27.17	-39.70	-10.69	-77.56
92237025	-14.87	-50.67	-11.87	-77.42
89442359	5.65	-47.26	-32.33	-73.94
23518335	-26.20	-41.68	-4.80	-72.69
71171599	-27.81	-39.52	-3.77	-71.10
189237	7.47	-51.25	-20.51	-64.29
92244160	-20.64	-18.15	-22.14	-60.93
102439955	-18.09	-30.85	-7.48	-56.42
10164932	4.24	-25.55	-28.28	-49.59
21910890	20.01	-54.13	-15.21	-49.33
10302154	-41.32	-4.54	-3.26	-49.12
126970231	-21.97	-20.89	-5.70	-48.56
53633108	-9.60	-18.94	-18.14	-46.68
3012941	5.16	-34.68	-10.40	-39.92
168311672	35.21	-16.73	-50.73	-32.25
92237026	-0.25	6.11	-35.42	-29.56
70911224	27.85	-27.93	-19.29	-19.36
92237032	12.29	14.77	-43.42	-16.35

Appendix E FEP Stage Results

Table E8: Results for the FEP stage in ALISE for the AlphaFold receptor model. The ligands are identified by their PubChem ID. The table contains free binding energy as well as the individual contributions to the binding free energy from the different steps of the thermodynamic cycle used in the FEP calculations.

**Ligand**	ΔG_1 **kcal/mol**	ΔG_2 **kcal/mol**	ΔG_3 **kcal/mol**	ΔG_4 **kcal/mol**	ΔG_5 **kcal/mol**	ΔG_0 **kcal/mol**
275781	22.49	22.40	0.00	32.35	7.58	-24.86
189237	18.14	32.15	0.00	37.03	3.96	-19.06
21910890	10.10	39.10	0.00	45.75	4.93	-11.83
132074004	15.43	44.66	0.00	39.60	3.71	-6.66
89442359	13.65	39.12	0.00	35.67	6.70	-3.50

Table E9: Results for the FEP stage in ALISE for the Hybrid receptor model. The ligands are identified by their PubChem ID. The table contains free binding energy as well as the individual contributions to the binding free energy from the different steps of the thermodynamic cycle used in the FEP calculations.

**Ligand**	ΔG_1 **kcal/mol**	ΔG_2 **kcal/mol**	ΔG_3 **kcal/mol**	ΔG_4 **kcal/mol**	ΔG_5 **kcal/mol**	ΔG_0 **kcal/mol**
132074004	19.73	51.64	0.00	62.07	5.89	-24.27
10302205	17.20	47.89	0.00	52.08	5.67	-15.72
154307377	13.69	26.84	0.00	21.91	5.13	-3.63
168270532	12.69	20.09	0.00	17.07	7.26	-2.42
21910880	14.77	40.71	0.00	34.08	7.75	-0.39

Table E10: Results for the FEP stage in ALISE for the Swiss receptor model. The ligands are identified by their PubChem ID. The table contains free binding energy as well as the individual contributions to the binding free energy from the different steps of the thermodynamic cycle used in the FEP calculations.

**Ligand**	ΔG_1 **kcal/mol**	ΔG_2 **kcal/mol**	ΔG_3 **kcal/mol**	ΔG_4 **kcal/mol**	ΔG_5 **kcal/mol**	ΔG_0 **kcal/mol**
21910880	26.64	42.56	0.00	48.63	9.06	-23.65
163033685	22.60	26.39	0.00	29.87	5.05	-21.02
88903284	13.25	50.15	0.00	60.27	7.23	-16.14
10302205	22.63	46.74	0.00	49.28	11.39	-13.78
77983075	13.87	20.09	0.00	21.28	10.58	-4.48

Appendix F Validation MD results

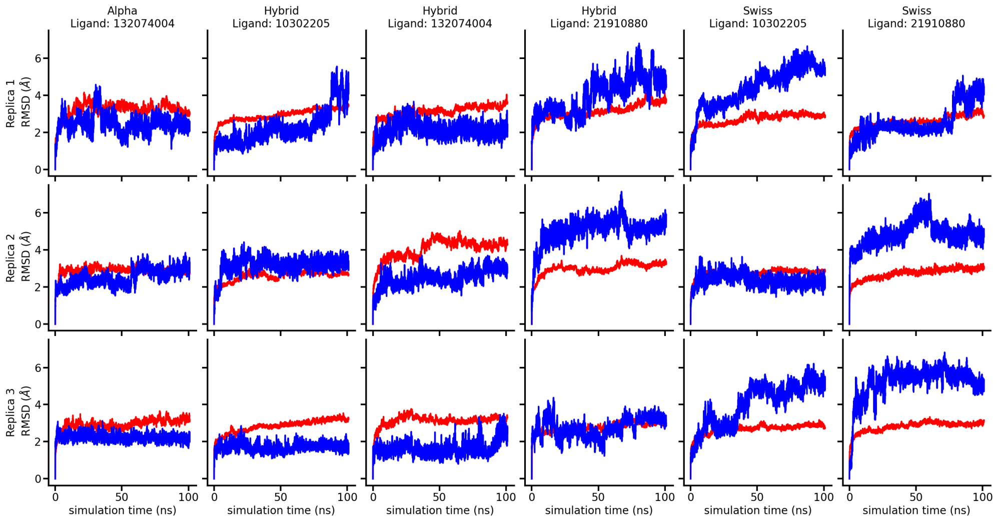

Fig. F11 In all six tested receptor-ligand combinations, the ligand stayed within its originally placed binding location over the period of 100 ns subjected to explicit solvent MD simulations (same parameters as FEP stage). The simulations have been aligned with respect to the backbone of the receptor, making the ligand completely free to move, such that its global translation would be captured by the RMSD. The low RMSD of below 7 Å indicates, that the ligand is not leaving the binding pocket in any of the studied cases.
